# Early Experiences in the Integration of Non-communicable Diseases into Emergency Primary Health Care, Beni Region, Democratic Republic of the Congo

**DOI:** 10.5334/aogh.3019

**Published:** 2021-03-19

**Authors:** Ruwan Ratnayake, Alison Wittcoff, John Majaribu, Jean-Pierre Nzweve, Lambert Katembo, Kambale Kasonia, Adelard Kalima Nzanzu, Lilian Kiapi, Pascal Ngoy

**Affiliations:** 1 Health Unit, International Rescue Committee, New York, NY, United States of America; 2 London School of Hygiene and Tropical Medicine, London, UK; 3 Health Unit, International Rescue Committee, New York, NY, United States of America; 4 International Rescue Committee, Goma, Nord Kivu, Democratic Republic of the Congo; 5 Division Provinciale de la Santé, Goma, Nord Kivu, Democratic Republic of the Congo; 6 Université Catholique du Graben et Université Officielle de Ruwenzori, Butembo, Democratic Republic of the Congo; 7 Health Unit, International Rescue Committee, London, United Kingdom

## Abstract

**Background:**

Health services in humanitarian crises increasingly integrate the management of non-communicable diseases into primary care. As there is little description of such programs, this case study aims to describe the initial implementation of non-communicable disease management within emergency primary care in the conflict-affected Beni Region of Democratic Republic of the Congo (DRC).

**Objectives:**

We implemented and evaluated a primary care approach to hypertension and diabetes management to assess the feasibility of patient monitoring, early clinical and programmatic outcomes, and costs, after seven months of care.

**Methods:**

We designed clinical and programmatic modules for diabetes and hypertension management for clinical officers and the use of patient cards and community health workers to improve adherence. We used cohort analysis (April to October 2018), time-trend analysis, semi-structured interviews, and costing to evaluate the program.

**Findings:**

Increases in consultations for hypertension (incidence rate ratio [IRR] 13.5, 95% CI 5.8–31.5, *p* < 0.00) and diabetes (IRR 3.6, 95% CI 1–12.9, *p* < 0.05) were demonstrated up to the onset of violence and an Ebola epidemic in August 2018. Of 833 patients, 67% were women of median age 56. Nearly all were hypertensives (88.7%) and newly diagnosed (95.9%). Treatment adherence, defined as attending ≥2 visits in the seven month period, was demonstrated by 45.4% of hypertension patients. Community health workers had contact with 3.2–3.8 patients per month. Respondents stated that diabetes care remained fragmented with insulin and laboratory testing located outside of primary care. Program and management costs were 115 USD per person per treatment course.

**Conclusions:**

In an active conflict setting, we demonstrated that non-communicable disease care can be well-organized through clinical training and cohort analysis, and adherence can be addressed using patient-held cards and monitoring by community health workers. Nearly all diagnoses were new, emphasizing the need to establish self-management. Insecurity reduced access for patients but care continued for a subset of patients during the Ebola epidemic.

## Introduction

In Africa, disability-adjusted life years due to non-communicable diseases (NCD) have increased by 67% between 1990 and 2017 [1]. While primary care remains the first point of contact across Africa, little is known about approaches to manage common NCDs, including hypertension and diabetes, through primary care [2]. Though mortality is primarily due to infectious, neonatal and maternal causes, the rising prevalence of NCDs and their risk factors, without a primary care backbone, requires attention [3]. In particular, the burden of diabetes and hypertension among crisis-affected populations is increasingly recognized, although arguably less emphasis has been placed on the substantial burden in Sub-Saharan Africa [4,5,6,7]. The estimated prevalence of diabetes in Democratic Republic of the Congo (DRC) ranges from 4% to 5.4% [8,9,10]. In Bukavu City and rural Katana in Sud Kivu, DRC, a near-exhaustive screening of households in 2016 found high prevalence of hypertension (18%) and obesity (9.8%) with low proportions under treatment (14%) or under control (43.5%) [11].

The challenges to providing continuous NCD care in unstable and conflict-affected settings are multiple: an obscure local epidemiological picture; limited infrastructure (including medications, equipment, and human resources); lack of familiarity with NCD management among providers; limited financing; risk of interruption resulting from insecurity or forced migration; and, the need to prioritize immediate infectious, maternal and neonatal health needs [8,12,13]. Practitioners have argued that NCD care during crises should focus on high-burden diseases (e.g., hypertension, diabetes mellitus, chronic obstructive pulmonary disorder, asthma); type 1 diabetes due to its severe outcomes; secondary prevention of severe disease and complications; and community health approaches for out-of-clinic monitoring and referral [4,14]. Psychiatric co-morbidities such as post-traumatic stress disorder may also impact motivation to seek care, and adherence to treatment [15,16].

While Médecins Sans Frontières has studied diabetes care delivered in a hospital in South Kivu, DRC in terms of perceived effects and continuity of care, there is a paucity of such detailed examination of the integration of NCD management into primary care [8,17]. The International Rescue Committee (IRC) has supported health care in DRC since 1996. In 2016, the IRC noted a rise in the burden of diabetes and hypertension cases in patient registers in Nord Kivu and proposed to pilot the integration of hypertension and diabetes into its primary care programs in crisis-affected Nord and Sud Kivu. In this study, we carried out a situational assessment of the initial implementation of NCD care delivered through primary care during a crisis in order to assess its feasibility in terms of monitoring and costs [14], and to capture the early clinical and programmatic outcomes after seven months of care. The assessment was used to guide programmatic decisions around the NCD program.

## Methods

### Study setting and population

Beni region is located in Nord Kivu, on the border with Orientale Province and Uganda. Since August 2017, the IRC has supported five primary care facilities in the Oicha and Mutwanga health zones. The catchment area includes the surrounding rural and remote areas and excludes Beni City. It has a population of 74,060 internally displaced persons (IDPs) and residents ≥18 years who use these clinics. Crude mortality across DRC and particularly in the Kivu provinces has been elevated for many years, due to infectious diseases and neonatal deaths stemming from persistent civil conflict [18]. Beni is continuously affected by conflict and its resulting population displacement. In 2018, the Allied Democratic Forces carried out attacks against civilians and peacekeepers, leading to pervasive insecurity and difficulties for movement [19]. To add to this complexity, an Ebola epidemic was declared in North Kivu in August 2018 and spread across Ituri and Sud Kivu [20]. Its containment has been deeply affected by insecurity and mistrust of authorities [21].

DRC’s health system relies primarily on primary care at the district level, which offers a basic package of essential care, and integrates reference centers which can perform additional laboratory services. At the secondary level, district hospitals provide inpatient and reference services, though they tend to also duplicate primary care services at cost [22]. The main challenges to the health system remain insufficient funding and predominance of user fees, overstaffing in urban versus rural health facilities, constrained provider capacity, and poor quality of health services [22]. Basic services for NCDs (e.g., provision of insulin and anti-hypertensives) are provided at the secondary level, but at-cost. Primary care staff have access to national protocols but do not receive in-service training for NCD management. Primary care facilities are typically staffed by clinical officers and nurses.

### Design of the integrated NCD program

To provide the foundation of NCD management through primary care, we added hypertension and type 1 and 2 diabetes to the primary care package, with disease selection based on the burden demonstrated in patient registers. The objective of the simplified protocol was to diagnose and maintain the health status of hypertension patients; maintain the health status of diabetes patients with known diagnoses; offer counselling and monthly follow-up; conduct simple cohort monitoring; and position community health workers (CHW) to offer community education on NCDs, counselling on diet and follow-up among cases in the community, and monitoring for adherence to medication and referral due to complications (see ***Box 2*** for a description of the service package). All adult patients were screened at each consultation using the case definitions outlined in ***Box 1***. Clinical advisors at the IRC (LK, PN) reviewed and adapted case management approaches from national and global guidelines (e.g., WHO and Primary Care International [PCI] guidelines; DRC Ministry of Health algorithms) into a curriculum with job-aids [6,23,24,25]. Clinical advisors from the Ministry of Health and the IRC (KK, KN, PN, including an endocrinologist and cardiologist) trained and evaluated clinical officers. A nurse/research coordinator (JM) met with the in-charge clinical officer at each health facility to setup the systems. Supervision visits to observe clinical care and enter data in the cohort monitoring system were conducted at least twice per month in the first month of implementation and once per month following.

**Box 1 Case definitions and management approaches for hypertension and diabetes, DRC, 2018**

**Table:** 


**Hypertension:** Adults ≥18 years with systolic blood pressure ≥140/90 mmHg and/or currently on hypertension medication.	**Diabetes:** Adults ≥18 years with a random blood glucose ≥200 mg/dl and/or currently on diabetes medication or insulin.

**Management approaches:** Diet and lifestyle advice. Medications: bisoprolol, enalapril, hydrochlorothiazide, nifedipine, methyldopa (essential hypertension), furosemide injectable if pulmonary edema.	**Management approaches:** Diet and lifestyle advice. Medications: glibenclamide, metformin, glucagon, glucose injectable (hypoglycemia), insulin (soluble, intermediate, rapid human).

**Box 2 Components of the integrated non-communicable disease program, DRC, 2018**

**Table:** 


**Clinical care**

**Clinical training of clinical officers in diagnosis and case management.** A French-language curriculum based on Primary Care International, WHO, and Ministry of Health guidelines were developed. The Ministry of Health and IRC clinical advisors delivered the training over a week. Theoretical topics included the physiology of diabetes and hypertension and practical topics included diagnosis, fundoscopy, clinical and home-based management, complications including diabetic foot, gestational diabetes, cardiovascular risk assessment, cardiac emergencies, and management among displaced populations.
**Drugs, consumables, and equipment supply.** Hypertensive and oral diabetes medications, insulin, and equipment (sphygmomanometers, glucometers, glucose strips, weighing scales) and laboratory supplies (blood glucose, urine protein/ketone strips) were provided.
**Referral costs to secondary care.** Given that primary care facilities are not expected to manage complications of hypertension and diabetes (e.g., diabetic foot), referral costs to the district hospital were covered by the IRC.
**Health facility assessment.** Using the WHO Package of Essential Noncommunicable Disease Interventions health facility assessment tool, a baseline assessment of readiness for NCD management was undertaken [23].

**Adherence and monitoring of care**

**Training and monitoring tools for CHWs.** CHWs were trained to provide community education sessions to describe available NCD services, symptoms and complications, danger signs, and dietary advice for patients. They were also trained to provide monthly household visits to known patients to monitor availability of medications, adherence to medications and clinic visits, and provide referrals if necessary.
**Cohort monitoring system.** Condensed essential NCD reporting to monitor programmatic outcomes of continuous care was implemented using a data entry and visualization program (EpiInfo7, CDC, Atlanta, GA, USA) on the research nurse’s laptop to perform cohort monitoring. Information was extracted from the patient register to the program on blood pressure (BP, mmHg), random blood sugar (RBS, mg/dL) for diabetics, patient outcome (remaining in care, died of and cause, transferred out, no monthly attendance, lost to follow up/defaulted after 90 days) and complications (defined as blindness, end-stage renal failure, myocardial infarction, congestive heart failure, stroke and/or above-ankle amputation) on a monthly basis. A dashboard self-populated with these program metrics. On a monthly basis, a report was produced for each health facility detailing the proportion of patients continuing care or absent, and under disease control. This report was discussed during a monthly meeting in each health facility.
This was paired with patient-held cards which were provided to the patient information on their diagnoses, medications, and date of next visit.

The project was approved by the Ministry of Health National Ethics Committee and the IRC’s institutional review board. Informed consent from interview respondents was documented. For the cohort analysis, since routine data was used, informed consent was not required.

### Evaluation methods

Cohort analysis, time-trend analysis, semi-structured interviews, and costing were undertaken. The cohort analysis, over the seven month program period (April to October 2018), produced monthly programmatic indicators for death, defaulting, and treatment outcomes using clinic visit data [26]. A time-trend analysis of consultations for hypertension and diabetes was undertaken over an extended two-year period (August 2017 to May 2019) to examine the uptake of services and the impact of violence and the onset of the Ebola outbreak (August 2018) [27]. During the study period (April to October 2018), monthly consultation rates and attendance among patients with ≥2 months of follow-up time served as a marker of adherence to care. The proportion of patients attending in September/October 2018 at target disease control (blood pressure ≤ 140/90 mmHg and/or random blood sugar ≤ 200 mg/dL) served as a marker for effectiveness of care. Adult contacts by CHWs and referrals by CHWs were assessed to evaluate the feasibility of using CHWs for out-of-clinic care.

Semi-structured interviews were carried out by the nurse-research coordinator (JM) in the local dialects and/or French before the program’s initiation and at the end of the study period, to gain insight into challenges for NCD management from the perspectives of clinical officers and patients. The interview guide captured issues affecting patients (e.g., barriers to accessing care and medications, achieving continuous care, referral, addressing risk factors, perceived benefits of care, common out of pocket costs of care) and health workers (e.g., low knowledge base, self-efficacy, barriers to quality care, and the ancillary services needed). Ideas for improvement were queried. Purposive sampling was used to achieve maximum variation in terms of demographics, role (for HCWs), and conditions (patients). Following transcription, thematic analysis using inductive coding was conducted by a single coder (RR) [28]. To improve trustworthiness, the preliminary qualitative findings were discussed with, and co-interpreted by, other investigators who were involved in the NCD program and interviews (JM, AW).

A cost analysis produced a cost efficiency estimate which compared the costs of a program per patient treated relative to one year of NCD care [29]. Direct costs per output were analyzed by integrating data on the output (number of cases treated) and individual program costs from budget expenditures including supplies (i.e., medications, medical equipment), and start-up activities (i.e., clinical training, CHW training, training materials, technical support and travel from health advisors). A second estimate combined direct costs with indirect support costs that were shared across IRC programs (i.e., shared personnel, equipment across programs). The two estimates were compared to estimates from other humanitarian health services delivered by the IRC in similar contexts.

## Results

### Initiation of services and context

Clinical officers were rapidly trained in March 2018, NCD clinical services were initiated in April 2018, medication stocks for acute presentations were in place in May 2018, and CHW support was added in July 2018. Insulin was kept at the district health office, given the need for refrigeration in the vaccine fridge. Therefore, while oral medications were available for patients with known diagnoses, diagnosis and management (using insulin) for diabetes could not be fully supported through IRC-supported facilities. From July 2018 to September 2018, attacks by the ADF resulted in civilian deaths, the burning of houses, and blockage of road access to Oicha health zone, at times preventing patients and personnel from accessing health facilities [19]. The Ebola outbreak, declared in August 2018, was rooted in Beni region, with 84% of the 279 suspected and confirmed cases by November 1, 2018 occurring there [30].

### Program outcomes

**Figure 1:**
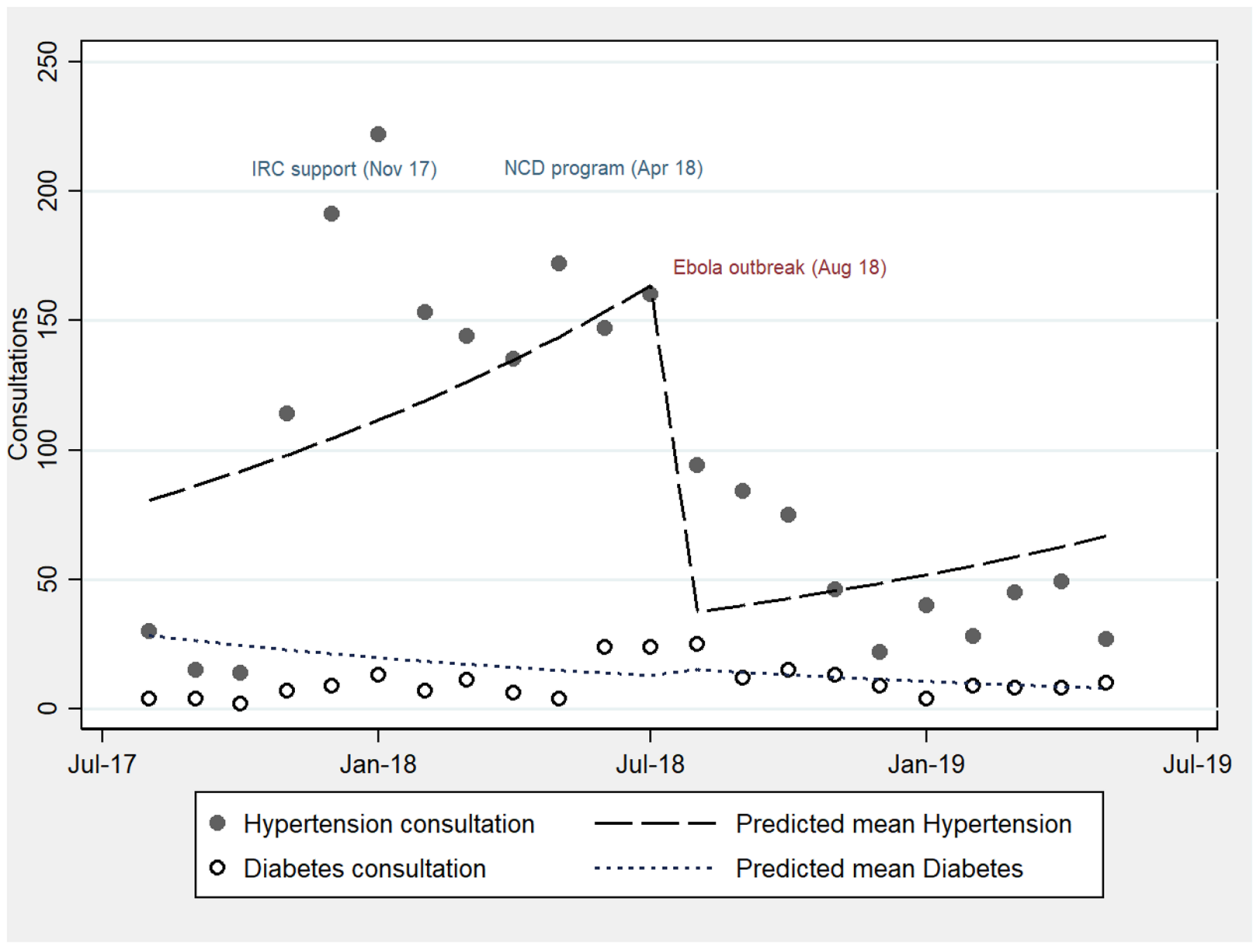
Counts and predicted means of hypertension and diabetes consultations, Aug 2017 to May 2019.

The monthly consultations for hypertension and diabetes and predicted mean trends were plotted across three periods: pre-program (August 1, 2017 to March 31, 2018); program initiation to the onset of Ebola and violence (April 1, 2018 to August 30, 2018), and post-disruption (September 1, 2018 to May 30, 2019) (***Figure 1***). IRC’s general support to primary care in Ministry of Health facilities started in November 2017 and the integration of NCDs in April 2018; this resulted in an overall increase in consultations for hypertension (incidence rate ratio [IRR] 13.5, 95% CI 5.8–31.5, *p* = 0.00) and diabetes (IRR 3.6, 95% CI 1–12.9, *p* = 0.046). Of note, slightly before the start of the NCD program, there were two months (December 2017 and January 2018) of high numbers of hypertension consultations. This is explained by increased attendance at a single reference center where hypertension medications were available; these numbers however were not sustained in the two subsequent months, and not observed in other centers. The onset of the NCD program appears to have elevated the hypertension and diabetes consultations, producing a continuous increase in the predicted mean trend. At the onset of Ebola and violence in August 2018, consultations decreased for hypertension (IRR 0.2, 95% CI 0.1–0.5, *p* < 0.001), with no change in trends for diabetes. From July to October 2018, CHWs had median 3.5 contacts (range, 3.2–3.8) with NCD patients per month. CHWs referred median 25.5 (range, 21.7–33.7) persons for diagnosis or care and monitored median 9.2 contacts (range, 8.7–11.2) with diagnosed patients per month.

The characteristics of 833 patients, recorded at registration, are described in ***Table 1***. The median age of patients was 56 years (IQR, 44–66). Most patients were newly diagnosed (798/833, 95.6%). Median body mass index (BMI) was 22.6 kg/m^2^ (IQR, 20.8–24.1); few patients were overweight or obese (134/833, 16.1%) and 5% were underweight. Forty-three percent of patients were displaced. Hypertensives (738/833, 88.6%), and few type I or II diabetics (57/833, 6.8%) or patients with both conditions (BC) (35/833, 4.2%) were registered. Among hypertensives, mean systolic blood pressure (SBP) was 161.1 mmHg (SD 22.4) and among diabetics, mean random blood sugar (RBS) was 290.5 mmol/L (SD 121).

**Table 1:** Characteristics of patients at registration (up to October 2018), N = 833.


CHARA CTERISTICS	ALL (N, %)	HYPERTENSION (N, %)	DIABETES (N, %)	BOTH CONDITIONS (N, %)

Cases (%, of all conditions)	833 (100)	739 (88.7)	59 (7.1)	35 (4.2)

Age*, median, IQR	56 (44–66)	56 (44–66)	55 (41–65)	60 (50–64)

Age group*				

<20	14 (1.7)	13 (1.8)	1 (1.7)	0

20–39	117 (14.2)	104 (14)	12 (20.3)	1 (2.9)

40–59	328 (39.9)	290 (39.2)	22 (37.3)	16 (45.7)

60 and above	374 (44.2)	321 (43.4)	24 (40.7)	18 (51.4)

Sex (female)	556 (66.8)	505 (68.3)	27 (45.8)	24 (68.6)

Displaced	364 (43.7)	342 (46.3)	11 (18.6)	11 (31.4)

Body mass index (BMI), median, IQR	22.6 (20.8–24.1)	22.6 (20.8–24.1)	22.2 (21–23.4)	22.6 (21.3–26.8)

Body mass index (BMI)				

Underweight	42 (5)	32 (4.3)	7 (11.9)	3 (8.6)

Normal	657 (78.9)	590 (79.8)	44 (74.6)	23 (65.7)

Overweight	118 (14.2)	103 (13.9)	7 (11.9)	8 (22.9)

Obese	16 (1.9)	14 (1.9)	1(1.7)	1 (2.9)

Newly-diagnosed	798 (95.9)	719 (97.3)	48 (82.8)	31 (88.6)

Systolic blood pressure* (SBP, mmHg), mean, SD	160, 25	161.1, 22.4	131.8, 53.3	159.9, 30.2

Random blood sugar* (RBS, mg/dl), mean, SD	–	–	290.5, 121.7	–


* Missing 11 ages, 36 SBP measures, 38 RBS measures (diabetes or both conditions).

Intermediate outcomes for all patients and patients remaining in care until September or October 2018 are listed in ***Table 2***. Adequate treatment adherence, defined as attending ≥2 visits where counselling, medications and/or insulin were received, was demonstrated by 45.4% (hypertension), 55.3% (diabetes), and 82.1% (both conditions) patients. Displaced persons (51.6%) compared to the host population (43.9%) were slightly more likely to demonstrate adequate treatment adherence (χ^2^ = 4.6, *p* = 0.03), while age, sex, and disease showed no significant observed differences. 254 (30%) of 833 patients remained in care (34%, hypertension; 50% diabetes; 56% [both conditions]). Disease control was demonstrated by 71% (hypertension), 60% (diabetes), and 50% (both conditions) of these patients. Few complications were documented.

**Table 2:** Attendance if registered by September 2018 (n = 788) and early outcomes if attended in September or October 2018 (n = 254).


OUTCOME	HYPERTENSION n/N (%)	DIABETES n/N (%)	BOTH CONDITIONS n/N (%)

Attended at least two visits^1^ (n = 373/788 or 47.3%)	324/713 (45.4)	26/47 (55.3)	23/28 (82.1)

Remained in care^2^ (n = 254/833 or 30.5%)	220/649 (33.9)	20/40 (50)	14/25 (56)

Outcome at last visit (remained in care only, N = 254)			

	*N = 220*	*N = 20*	*N = 14*

SBP* (mmHg) (mean, SD)	133, 12.7	–	136.8, 18.9

SBP* <140 mmHg	157 (71.4)	–	8 (57.1)

RBS* (mg/dL) (mean, SD)	–	219.4, 79.6	187.1, 63.2

RBS* ≤200 mg/dL	–	12 (60)	7 (50)

Complications^3^	11 (5)	1 (5)	1 (7)

Died	1 (<1)	1 (5)	–


^1^ Patients eligible for at least two visits during the time period (registered by September 2018); ^2^ Patients attended the September or October visit (registered by August 2018); ^3^ Complications included diabetic foot, gestational diabetes, cardiovascular risk assessment, cardiac emergencies; SBP, systolic blood pressure; RBS, random blood sugar; SD, standard deviation. * Missing 16 SBP values, 5 RBS values.

### Challenges and improvements to NCD care

Five patients from three health facilities and five HCW from four health facilities were interviewed at baseline; seven patients and six HCW from three health facilities were interviewed at endline. The major challenges voiced by patients at baseline were related to poor access due to the costs of transport for monthly visits and medications, and fragmentation of diabetes care between primary care, laboratories and district health offices which store insulin. HCWs mentioned the gaps across the spectrum of care in training, diagnostics, access to treatment, health information systems to continually monitor patients, and referral and counter-referral systems (Panel 1, ***Box 3***). At endline, patients and HCWs mentioned several positive factors about the NCD integration, including the linkage of patients with CHWs, and coordination of program elements (Panel 2). Several enduring challenges were noted by HCWs, relating to the quality and comprehensiveness of care (particularly for diabetes), reliance on key equipment, and sustainability of prevention among patients (Panel 3).

**Box 3 Semi-structured interview quotes**

**Table:** 


**PANEL 1: CHALLENGES TO NCD CARE (BASELINE).**

*We don’t have money to get [to the facilities] and to get care [i.e. drugs and insulin]. Money is limited when there is little farming happening in the fields*. (Patient) *I take [medications] when problems present themselves and I stop afterwards [because of the costs]*. (Patient) *[Communities] are aware of NCDs but [health facilities] are limited [in our response] because we lack medications. Communities become more serious about communicable diseases that kill rapidly than non-communicable diseases which kill slowly*. (HCW)

**PANEL 2: IMPROVEMENTS TO NCD CARE (ENDLINE).**

*When we removed the costs, the patients come directly and consistently. We even get patients from Mutwanga health zone, here in Bulongo. That’s 10 km away*. (HCW) *We have one day a week, Thursdays, to see all patients with non-communicable diseases. The day is well known in the community*. (HCW) *Even yesterday, a CHW came to remind me of my appointment. They can follow-up with things at the health facility, sensitize people, motivate people to respect the appointments*. (Patient)

**PANEL 3: ENDURING CHALLENGES (ENDLINE).**

*Community sensitization may be there, but [the patients and community] will ignore it. It is difficult to follow advice [on primary and secondary prevention]*. (HCW) *Diabetes cases are [still just as] frequently referred to the general hospital as [in primary care]. We just don’t have the appropriate medications and materials*. (HCW) *Each patient needs a drug plan addressed [specifically] for them [and this is a lot of work and resources]*. (HCW) CHW, community health worker; HCW, health care worker.

### Costing of the program

Direct costs including medications, medical equipment, start-up trainings, and technical support produced an estimate of 67 USD per person per treatment course. Accounting for both direct and indirect shared costs (i.e., shared personnel and equipment used across IRC), the costs increased to 115 USD per person per treatment course. The highest proportions of program costs were borne by medications, equipment, and trainings (36% of total costs) and national staff salaries (11.7%).

## Discussion

In this situational assessment of a simplified protocol for NCD integration into emergency primary care, we demonstrated that the rapid training and integration of hypertension and diabetes care and patient monitoring into an ongoing emergency primary care in Beni Region, DRC is feasible. WHO estimates ≤50% of patients in low and middle-income countries adhere to long-term therapies for chronic diseases including NCDs [31]. If health care utilization (where medication is dispensed) is used as a crude proxy measure for medication adherence, that 45% of hypertensives attended ≥2 clinic visits during a seven month period suggests substantial room for improvement. It also may reflect the frequency of care that could be expected in a conflict area where patients have unpredictable accessibility [13]. The NCD program’s addition of a consistent supply of medications and start-up training and resources into primary care appears to have acted to sustain a consistent number of consultations for at least four months, as compared to sporadic attendance earlier. Violence in Oicha health zone effectively reduced access to care but care continued for a subset of patients throughout the initial phase of the Ebola outbreak and insecurity. Care was well-organized through the use of clinical guidelines and cohort monitoring. Adherence was addressed through patient-held cards and CHW monitoring at a frequency that appeared feasible with their existing workload. A meaningful proportion of hypertensives remained under control (71%). At 67/115 USD (program/total costs) per person per treatment course per year, hypertension and diabetes care in DRC was 50% lower than the costs per patient for hospital-based diabetes care in South Kivu, DRC (~230 USD) and more comparable with costs per patient for the community management of acute malnutrition programs in East and West Africa (Niger [98/142 USD], Mali [105/120 USD], Kenya [112/119]) [8,32].

We learned that the organization of primary care services to address diabetes care has additional complexity. First, the patient profile was weighted toward a risk group (adults 40 years and older, hypertensives, and displaced persons), but also revealed the absence of diabetics seeking primary care. Respondents stated that diabetes care was still fragmented offering little to be done at the primary care level. Diabetes laboratory testing was frequently unavailable, and traveling to the district health office to obtain insulin, after attending primary care, remains a difficult cycle for the patient to achieve. This issue of fragmentation has been shown to be a key obstacle in other settings, notably in stable and more advanced primary care systems in rural India [33]. Other studies in Sud Kivu have cited a particularly low awareness of diabetes, its symptoms, and available care, emphasizing the need for community sensitization and mobilization for care, through CHWs [17]. Second, nearly all diagnoses were new, emphasizing the need for concurrent counselling and self-management support to establish self-care among new patients and those who are forcibly displaced [34]. This requires additional dedicated support for counseling in the clinic, to supplement the clinical visit. Health worker quotes reflecting that “each patient needs a drug plan” and the difficulty of following counseling advice given available food and monetary resources further reflects that it is unlikely that drug treatment alone can lead to sustained disease control. Complications, like diabetic foot, were either not well-recognized by the recently-trained clinical officers, or patients with complications may have gone directly to secondary care. A care and referral plan for each complication should therefore be developed ahead of time, rather than proposing general referral for all complications (many of which simply cannot be addressed in a disrupted health system).

The adherence and monitoring aspects of the intervention appear foundational for achieving continuous care. First, the cohort analysis approach succeeded in describing the burden of disease at the health facility level in a more action-oriented manner [35]. However, recording monthly data in near real-time in an EpiInfo7 database was not as efficient as intended due to the reliance on the nurse coordinator. A registry compiled each day or week would be less onerous and could provide equally-timely analysis. Second, CHW programs targeting out-of-clinic improvements of adherence to medications, and reminders of clinical visits have been shown to be effective in disease control and medication adherence in LMICs [36,37]. In DRC, CHWs had a manageable workload in terms of household visits to NCD patients and community awareness-raising. However, during an emergency, CHWs may be more efficiently used to monitor a subset of the NCD patients at high-risk of decompensation (e.g., children and adults with type I diabetes), those with comorbidities and complications, and elderly and disabled patients with limited mobility [13].

The evaluation was limited by the difficulty of achieving complete and timely data collection among a clinical cohort in a conflict area. As well, we did not undertake quality of care assessments, aside from examining in-range blood pressure and blood sugar values. This leaves open the question of the effectiveness of this curriculum in providing adequate-quality care. The short follow-up period and lack of control group precluded an examination of the effects of therapy over time. Collecting information on self-reported comorbidities would be useful for stratifying patients by level of risk. Finally, there was little insight gained into the appropriateness and impact of the dietary education, considering the food-insecure context.

Overall, this study showed that the integration of NCD care into emergency primary care is feasible in the short term, if the health system strengthening, particularly strong service delivery, workforce trained in NCD, HIS, access to essential medicines, and financing can be met. The IRC continued to support patients with medications and consultations free of charge, and used the results to improve adherence of patients to clinic visits through the linkage with the CHW program. Enduring gaps include providing professional staff to provide psychosocial care, and mobilizing dietary support for diabetics.
